# Ethnic differences in oral health and use of dental services: cross-sectional study using the 2009 Adult Dental Health Survey

**DOI:** 10.1186/s12903-016-0228-6

**Published:** 2016-06-16

**Authors:** Garima Arora, Daniel F. Mackay, David I. Conway, Jill P. Pell

**Affiliations:** Institute of Health and Wellbeing, University of Glasgow, 1 Lilybank Gardens, Glasgow, G12 8RZ UK; Dental School, University of Glasgow, 378 Sauchiehall Street, Glasgow, G2 3JZ UK

**Keywords:** Dental health services, Ethnic groups, Oral health, Survey, Dental health

## Abstract

**Background:**

Oral health impacts on general health and quality of life, and oral diseases are the most common non-communicable diseases worldwide. Non-White ethnic groups account for an increasing proportion of the UK population. This study explores whether there are ethnic differences in oral health and whether these are explained by differences in sociodemographic or lifestyle factors, or use of dental services.

**Methods:**

We used the Adult Dental Health Survey 2009 to conduct a cross-sectional study of the adult general population in England, Wales and Northern Ireland. Ethnic groups were compared in terms of oral health, lifestyle and use of dental services. Logistic regression analyses were used to determine whether ethnic differences in fillings, extractions and missing teeth persisted after adjustment for potential sociodemographic confounders and whether they were explained by lifestyle or dental service mediators.

**Results:**

The study comprised 10,435 (94.6 %) White, 272 (2.5 %) Indian, 165 (1.5 %) Pakistani/Bangladeshi and 187 (1.7 %) Black participants. After adjusting for confounders, South Asian participants were significantly less likely, than White, to have fillings (Indian adjusted OR 0.25, 95 % CI 0.17-0.37; Pakistani/Bangladeshi adjusted OR 0.43, 95 % CI 0.26-0.69), dental extractions (Indian adjusted OR 0.33, 95 % CI 0.23-0.47; Pakistani/Bangladeshi adjusted OR 0.41, 95 % CI 0.26-0.63), and <20 teeth (Indian adjusted OR 0.31, 95 % CI 0.16-0.59; Pakistani/Bangladeshi adjusted OR 0.22, 95 % CI 0.08-0.57). They attended the dentist less frequently and were more likely to add sugar to hot drinks, but were significantly less likely to consume sweets and cakes. Adjustment for these attenuated the differences but they remained significant. Black participants had reduced risk of all outcomes but after adjustment for lifestyle the difference in fillings was attenuated, and extractions and tooth loss became non-significant.

**Conclusions:**

Contrary to most health inequalities, oral health was better among non-White groups, in spite of lower use of dental services. The differences could be partially explained by reported differences in dietary sugar.

## Background

Dental caries is a dynamic process of demineralization and remineralisation that is potentially reversible prior to the development of cavitation. In spite of this, untreated dental decay was the most common of the 301 diseases studied across 188 countries in the Global Burden of Disease Study [1]. Wide socioeconomic [2] and ethnic [3] inequalities have been reported in both the prevalence and severity of oral diseases.

The consumption of sugar has recently been recognized by the World Health Organisation as a major public health challenge and a common risk factor for many chronic diseases [4]. The ingestion of sugar, contained in confectionary, foods [5–7], some medicines [8] and carbonated, soft drinks [9], predisposes to the demineralization of enamel and, therefore, dental caries. Poor plaque control predisposes to periodontitis which is also associated with tobacco smoking [10], and emerging risk factors including heavy alcohol consumption [11] and nutritional deficiencies [12]. Lifestyle risk factors, such as smoking [13] and dietary sugar [14], are known to vary by ethnic group.

Uncontrolled dental caries and periodontal disease are the main causes of tooth loss which has been consistently associated with poor health [10]. Oral health is a prerequisite of good general health and quality of life. Poor oral health can result in pain and difficulties eating or speaking which, in turn, can restrict everyday life including employment [15]. Moreover, the societal economic burden is considerable, with the treatment of oral diseases consuming 5-10 % of health service budgets in high income countries [16].

Whilst sugar causes dental caries and, therefore, subsequent outcomes such as fillings, extractions and tooth loss, these associations can be moderated by other factors such as dental hygiene, fluoridation and consumption of acidic drinks. Therefore, differences in oral health between sub-groups of the populations could be driven by a range of factors.

In the United Kingdom (UK), ethnic minority groups account for an increasing percentage of the general population; non-White groups accounted for 13 % of the UK population in the 2011 Census [17]. In London, the most ethnically diverse UK city, only 46 % of the population were classified as White British in the last Census [17]. Therefore, understanding ethnic differences is an important aspect to understanding the population’s general and oral health and to planning appropriate, responsive services. However, ethnic differences in oral health have been relatively neglected in comparison to the substantial literature on socioeconomic inequalities in oral health and access to dental services [2, 18].

Self-classification of ethnicity is a complex paradigm that encompasses many facets: ancestral, geographical, genetic and physical characteristics as well as religion, language, culture and custom. Ethnicity impacts on both health beliefs and health behaviours; including beliefs and behaviours pertinent to oral health. Therefore, ethnic groups vary in lifestyle, such as alcohol consumption, smoking and diet, as well as their use of preventive and treatment services; including dental services. Furthermore, the innate characteristics of ethnic groups interact with environmental influences such as: differences in individual- and area-based socioeconomic status [19]; the impacts of migration, assimilation and discrimination [20]; and the ethnic mix of the communities in which people reside. Previous studies suggest that, whilst much of the ethnic differences in health and oral health can be explained by socioeconomic factors, cultural and behavioural factors and access to health, including dental, services may also play a role [3, 21, 22].

Studies on adult oral health emanate mainly from the United States of America (USA) [3, 21], while European and UK epidemiological studies have tended to focus on child oral health outcomes [22]. However, care should be heeded in extrapolating findings from adult studies conducted in the USA to Europeans and UK populations, for example ethnicity is a much stronger proxy of socioeconomic status in the former. The aim of this study was to determine whether there are ethnic differences in adult oral health in the UK and, if so, whether these persist following adjustment for differences in sociodemographic factors, lifestyle, or use of dental services.

## Methods

The Adult Dental Health Survey (ADHS) is a cross sectional study that has been conducted every ten years since 1968 [23]. It was established to provide important information about the general population’s oral health and their access to, and experience of, dental services. The information gleaned is used to plan dental services and monitor adherence to governmental targets. The most recent survey was conducted in 2009 and covered England, Wales and Northern Ireland. In contrast to the two previous surveys, Scotland did not participate. The Oxford National Health Service (NHS) Research Ethics Committee provided ethical approval for the survey to be conducted and anonymised, individual-level data are freely accessible to registered researchers via the UK Data Service.

The 2009 ADHS used a two-stage cluster sampling design. In the first stage, 253 primary sampling units were identified in England and Wales and another 15 in Northern Ireland. Within these primary sampling units, two postcode sectors were then selected from which 25 addresses were sampled to give a total sample size of 13,400 households. These 13,400 households comprised 1,150 households from each of the ten English Strategic Health Authorities (mean population 5.4 million): 1,150 from Wales (population 3.1 million) and 750 from Northern Ireland (population 1.8 million). Recruitment was not stratified by ethnic group and there was no boosted sampling of ethnic minority groups. The household response rate was 60 %. Within participating households, all adults (>16 years) were invited to participate in a face to face interview and participants with at least one natural tooth were invited to undergo a subsequent dental examination. The examination was conducted by NHS salaried dentists who attended study training over four days, undertook supervised practice examinations of volunteers, and underwent a calibration exercise. In brief, the examination included assessment of the condition of teeth surfaces, root surfaces, spaces, aesthetics and dentures, as well as a basic periodontal examination to assess the periodontal condition [24]. Full details of the dental examination are contained in the survey technical report [25]. Of the 13,509 individuals invited to participate in the 2009 ADHS, 11,380 (84 %) consented to the interview and, of the 10,457 dentate interviewees, 6,469 (62 %) underwent the subsequent dental examinations. Both the interview and examination were conducted in participants’ homes.

Data were collected during two ten week periods: October to December 2009 and January to April 2010. The self-reported data collected during the interview and included in our analyses were: sociodemographic information (age, sex, ethnic group, household tenure, number of household members, and educational qualifications (yes/no)), lifestyle (dietary sugar intake and smoking status), use (type of service provider, frequency and type of attendance) and perception of dental services, personal dental hygiene (teeth cleaning with toothpaste and toothbrush, and use of other dental hygiene products including dental floss and mouthwash), dental history (fillings, extractions, dentures and problems eating), and self-reported overall oral health. Postcode of residence was used to derive the Index of Multiple Deprivation (IMD). This is an area-based measure of socioeconomic deprivation derived from 38 indicators collected across seven domains: income, employment, health deprivation and disability, education skills and training, barriers to housing and services, crime and living environment. It is derived at the lower layer super output level which equates to a mean population of 1,600 (range 1,000 to 3,000). The IMD is used to derive quintiles for the general population and the postcode of resident of survey participants could, therefore, be used to allocate participants to a general population socioeconomic deprivation quintile.

We excluded from the study participants who did not provide information on their ethnic group and those whose ethnic group was recorded as mixed or other, because the heterogeneity within these groups would render results difficult to interpret and generalize. Pakistani and Bangladeshi groups had to be combined due to small samples sizes, as did Black African and Caribbean. Therefore the groups included in the analyses were White, Indian, Pakistani/Bangladeshi and Black. The ethnic groups were compared in terms of demographics and socioeconomic status, lifestyle risk factors, use of dental services, personal dental hygiene and self-reported oral health using χ^2^ tests for categorical data. Missing data were imputed using imputation through chained equations using the user written “ice” module in Stata. Twenty imputed datasets were created. Logistic regression models, weighted using the ADHS provided weights, were developed for three separate outcomes available within the survey: presence of any fillings, history of any extractions and possession of fewer than 20 teeth (the recognized threshold for functional dentition) [26]. For each of these outcomes, the White group was used as the reference category. The models were run univariately and then multivariably adjusting for potential confounders (age, sex, educational qualifications, housing tenure, area socioeconomic deprivation quintile and area of residence). We then ran three further multivariable models, including additional covariates, to adjust for: potential lifestyle mediators, potential dental service mediators and both. Lifestyle mediator variables were: frequency of consumption of sweets, cakes and fizzy drinks, adding sugar to hot drinks, smoking status, frequency of teeth cleaning (≥ or < twice per day) and current use of dental hygiene products other than a manual toothbrush and toothpaste. Dental service mediators were: type of service provider (last dental service visit reported as NHS, private or other including hospital dental care), frequency of dental clinic visits (≥2 in past year, 1 in past year, 1 in past 2 years, <1 in past 2 years, or only if symptoms) and ever having had a scale and polish. All of the multivariable models produced areas under the receiver operating characteristic (ROC) of at least 76 % suggesting that the models were a good fit for the data.

We undertook additional logistic regression models in the dentate sub-group who underwent clinical examination. Because of smaller numbers of participants, we combined Indian, Pakistani and Bangladeshi participants into a single South Asian group and excluded Black participants. The two main outcomes studied were: presence of dental caries (defined as ≥1 tooth with untreated decay) and presence of periodontal pockets (defined as ≥1 pocket of ≥6 mm). As with the previous analyses, we used ADHS provided sample weights, ran the models univariately and then adjusted for confounders. We then added lifestyle and dental service mediators as covariates in two further models. The multivariable models all had areas under the receiver operator characteristic (ROC) of at least 0.63 suggesting the models did not adequately explain the variation in outcome variables observed in the dentate sub-group.

All analyses were undertaken using Stata version 14.1. Statistical significance was defined as *p* < 0.05. Statistical interactions could not be tested due to insufficient statistical power.

## Results

Of the 11,380 participants, 22 (0.2 %) were excluded because they had missing data on ethnic group. We excluded a further 299 participants whose ethnic group was recorded as other, Asian other or mixed. The remaining 11,059 participants comprised the study population. Of these: 10,435 (94.6 %) were White, 272 (2.5 %) Indian, 165 (1.5 %) Pakistani or Bangladeshi and 187 (1.7 %) Black. The sex breakdown was similar across ethnic groups but there were significant differences in other sociodemographic measures. White participants were significantly older and had fewer household members. They were less socioeconomically deprived, as measured by home ownership and area-based deprivation, but were less likely to have achieved formal educational qualifications (Table 1). Missing data for these sociodemographic variables was very low.

**Table 1:** Demographic characteristics by ethnic group

	White	Indian	Pakistani/Bangladeshi	Black	*p* value*
	*N* = 10,435	*N* = 272	*N* = 165	*N* = 187	
	*N* (%)	*N* (%)	*N* (%)	*N* (%)	
Sex					
Female	5,790 (55.5)	147 (54.0)	89 (53.9)	98 (52.4)	0.789
Male	4,645 (44.5)	125 (46.0)	76 (46.1)	89 (47.6)	
Age (years)					
16-34	2,166 (20.8)	101 (37.1)	68 (41.2)	69 (36.9)	<0.001
35-54	3,689 (35.4)	102 (37.5)	78 (47.3)	87 (46.5)	
≥55	4,580 (43.9)	69 (25.4)	19 (11.5)	31 (16.6)	
Number of household members					
1	1,924 (18.4)	18 (6.6)	8 (4.9)	48 (25.7)	<0.001
2	4,458 (42.7)	75 (27.6)	32 (19.4)	47 (25.1)	
3-5	3,864 (37.0)	141 (51.8)	90 (54.6)	76 (40.6)	
≥6	189 (1.8)	38 (14.0)	35 (21.2)	16 (8.6)	
Household tenure					
Owner occupied	9153 (87.8)	198 (72.8)	115 (69.7)	145 (77.5)	<0.001
Rented	1275 (12.2)	74 (27.2)	50 (30.3)	42 (22.5)	
Missing	7	0	0	0	
Socioeconomic deprivation quintile					
1 (most deprived)	1,438 (14.2)	46 (16.9)	69 (41.8)	102 (54.6)	
2	1,928 (18.5)	99 (36.4)	36 (21.8)	27 (14.4)	
3	2,330 (22.3)	69 (25.4)	43 (26.1)	28 (15.0)	
4	2,315 (22.2)	22 (8.1)	9 (5.5)	18 (9.6)	
5 (least deprived)	2,379 (22.8)	36 (13.2)	8 (4.9)	12 (6.4)	
Missing	0	0	0	0	<0.001
Educational qualifications					
Yes	7,370 (70.8)	213 (78.3)	117 (71.3)	142 (75.9)	
No	3,046 (29.2)	59 (21.7)	47 (28.7)	45 (24.1)	0.023
Missing	19	0	1	0	

N number

*χ^2^ test

Lifestyle risk factors also varied significantly by ethnic group with very few missing observations. White participants were least likely to add sugar to hot drinks but they consumed sweets and cakes significantly more frequently than participants from other ethnic groups and had the highest prevalence of smoking (Table 2). Black participants consumed sweets and cakes least frequently. However, they were the most frequent consumers of fizzy drinks.

**Table 2:** Lifestyle risk factors by ethnic group

	White	Indian	Pakistani/Bangladeshi	Black	*p* value^*^
	*N* = 10,435	*N* = 272	*N* = 165	*N* = 187	
	*N* (%)	*N* (%)	*N* (%)	*N* (%)	
Consumption of sweets					
Rarely/Never	1,695 (16.2)	60 (22.1)	46 (27.9)	63 (33.7)	<0.001
<1/week	1,751 (16.8)	45 (16.5)	23 (13.9)	47 (25.1)	
1-2/week	3,014 (28.9)	72 (26.5)	48 (29.1)	44 (23.5)	
3-5/week	2,211 (21.2)	56 (20.6)	21 (12.7)	27 (14.4)	
≥6/week	1,763 (16.9)	39 (14.3)	27 (16.4)	6 (3.2)	
Missing	1	0	0	0	
Consumption of cakes					
Rarely/Never	785 (7.5)	20 (7.4)	14 (8.5)	41 (21.9)	<0.001
<1/week	1,035 (9.9)	30 (11.0)	18 (10.9)	33 (17.7)	
1-2/week	2,670 (25.6)	113 (41.5)	48 (29.1)	63 (33.7)	
3-5/week	2,782 (26.7)	52 (19.1)	39 (23.6)	34 (18.2)	
≥6/week	3,162 (30.3)	57 (21.0)	46 (27.9)	16 (8.6)	
Missing	1	0	0	0	
Consumption of fizzy drinks					
Rarely/Never	3,859 (37.0)	80 (29.4)	37 (22.4)	28 (15.0)	<0.001
<1/week	1,125 (10.8)	36 (13.2)	13 (7.9)	27 (14.4)	
1-2/week	1,418 (13.6)	52 (19.1)	38 (23.0)	38 (20.3)	
3-5/week	1,190 (11.4)	39 (14.3)	26 (15.8)	37 (19.8)	
≥6/week	2,841 (27.2)	65 (23.9)	51 (30.9)	57 (30.5)	
Missing	2	0	0	0	
Add sugar to hot drinks					
No	6,746 (64.7)	95 (34.9)	47 (28.5)	67 (35.8)	<0.001
Yes	3,689 (35.4)	177 (65.1)	118 (71.5)	120 (64.2)	
Smoking Status					
Never	4,454 (42.7)	214 (78.7)	124 (75.2)	117 (62.6)	<0.001
Former	3,697 (35.5)	40 (14.7)	25 (15.2)	33 (17.7)	
Current	2,271 (21.8)	18 (6.6)	16 (9.7)	37 (19.8)	
Missing	13	0	0	0	

N number

*χ^2^ test

The last visit for dental services was most commonly provided by the NHS in all ethnic groups. Use of private sector dentists was most common among White participants, and least common among the combined Pakistani/Bangladeshi group (Table 3). There were no significant differences in the perception of dental services. Compared with White participants, South Asian participants were less likely to have had a scale and polish or use other dental hygiene products (such as dental floss or mouthwash); they attended routine dental clinic visits less frequently and were more likely to report that they only attended the dentist if they suffered symptoms. Indian participants also brushed their teeth less frequently. Compared with White participants, Black participants had less frequent dental clinic visits and were less likely to have had a scale and polish and to use other dental hygiene products. However, they brushed their teeth more frequently than White participants. Missing data on the use of dental services and preventative measures was higher than for demographic and lifestyle data and all ethnic groups recorded some missing data. Poor recall is a plausible reason for some missing data as not all patients will remember their last visit to the dentist; especially if visits are infrequent. Similarly, infrequent attenders may feel unable to rate the quality of their dental service.

**Table 3:** Use of dental services and dental preventative measures by ethnic group

	White	Indian	Pakistani/Bangladeshi	Black	*p* value*
	*N* = 10,435	*N* = 272	*N* = 165	*N* = 187	
	*N* (%)	*N* (%)	*N* (%)	*N* (%)	
Service provider					
Private	2,915 (28.3)	58 (21.3)	19 (11.5)	30 (16.0)	<0.001
NHS	7,136 (69.2)	165 (67.4)	125 (83.3)	126 (75.5)	
Other	267 (2.6)	22 (9.0)	6 (4.0)	11 (6.6)	
Missing	117	27	15	20	
Perception of dental services					
Very good/good	8,097 (89.1)	195 (87.1)	111 (84.1)	128 (86.5)	0.172
Fair/poor/very poor	994 (10.9)	29 (13.0)	21 (15.9)	20 (13.5)	
Missing	1,344	48	33	39	
Frequency of visits to dentist					
≥2 in 1 year	5,306 (51.5)	80 (32.3)	50 (32.9)	64 (38.1)	<0.001
1 in 1 year	2,057 (20.0)	48 (19.4)	25 (16.5)	30 (17.9)	
1 in 2 years	439 (4.3)	23 (9.3)	21 (13.8)	13 (7.7)	
<1 in 2 years	864 (8.4)	49 (19.8)	25 (16.5)	31 (18.5)	
Only if symptoms	1,639 (15.9)	48 (19.4)	31 (20.4)	30 (17.9)	
Missing	130	24	13	19	
Ever had scale and polish					
Yes	8,513 (82.6)	162 (65.6)	108 (71.5)	109 (64.5)	<0.001
No	1,795 (17.4)	85 (34.4)	43 (28.5)	60 (35.5)	
Missing	127	25	14	18	
Frequency of teeth cleaning					
≥ twice daily	7,228 (75.0)	170 (64.2)	124 (75.2)	144 (81.0)	<0.001
< twice daily	2,411 (25.0)	95 (35.9)	41 (24.9)	33 (18.6)	
Missing	796	7	0	10	
Use other dental hygiene products					
Yes	6,083 (58.8)	117 (43.3)	61 (37.0)	81 (43.6)	<0.001
No	4,265 (41.2)	153 (56.7)	104 (63.0)	105 (56.5)	
Missing	87	2	0	1	

N number of participants

*χ^2^ test

White and Indian participants were more likely to rate their own oral health as either good or very good, and were the least likely to report difficulties eating due to dental problems (Table 4). In spite of this, White participants were the most likely to report missing teeth, fillings or dentures. Almost half of Pakistani/Bangladeshi participants rated their oral health as bad or poor in spite of being least likely to report missing teeth and dentures.

**Table 4:** Self-reported oral health by ethnic group

	White	Indian	Pakistani/Bangladeshi	Black	*p* value^*^
	*N* = 10,435	*N* = 272	*N* = 165	*N* = 187	
	*N* (%)	*N* (%)	*N* (%)	*N* (%)	
Overall oral health					
Very good/good	7,471 (71.7)	198 (72.8)	92 (55.8)	128 (68.0)	<0.001
Bad/poor	2,952 (28.3)	74 (27.2)	73 (44.2)	59 (31.6)	
Missing	12	0	0	0	
Natural teeth					
None	794 (7.6)	7 (2.6)	0	10 (5.4)	<0.001
1-9	528 (5.1)	5 (1.8)	3 (1.8)	5 (2.7)	
10-19	1,248 (12.0)	9 (3.3)	4 (2.4)	11 (5.9)	
≥20	7,836 (75.3)	251 (92.3)	157 (95.7)	160 (86.0)	
Missing	29	0	1	1	
Any fillings					
Yes	8,434 (87.5)	161 (61.0)	102 (61.8)	107 (60.8)	<0.001
No	1,201 (12.5)	103 (39.0)	63 (38.2)	69 (39.2)	
Missing	800	8	0	11	
Denture					
Yes	2,472 (23.7)	20 (7.4)	10 (6.1)	34 (18.2)	<0.001
No	7,962 (76.3)	252 (92.7)	155 (93.9)	153 (81.8)	
Missing	1	0	0	0	
Difficulty eating due to dental problems					
Yes	2,156 (20.7)	65 (23.9)	47 (28.7)	56 (29.9)	
No	8,277 (79.3)	207 (76.1)	117 (71.3)	131 (70.1)	0.002
Missing	2	0	1	0	

N number

* χ^2^ test

After adjusting for the potential confounding effects of age, sex, educational qualifications, household tenure, area socioeconomic deprivation quintile and area of residence, South Asian participants remained significantly less likely, than White participants, to report fillings, dental extractions and having less than 20 teeth (Table 5). Adjustment for use of dental services (type of dental service provider, frequency of dental appointments and scale or polish) attenuated the lower risk of fillings among Indian participants but it remained significant. Addition of potential lifestyle mediators (frequency of consumption of sweets, cakes and fizzy drinks, adding sugar to hot drinks, smoking status, frequency of teeth cleaning, and use of other dental hygiene products) to the model attenuated the differences between South Asian and White participants, but the associations remained statistically significant.

**Table 5:** Logistic regression analysis of the association between ethnic group and oral health

	Univariate		Multivariate adjusted for confounders^a^		Multivariate adjusted for confounders^a^ & lifestyle mediators^b^		Multivariate adjusted for confounders^a^ & dental service mediators^c^		Multivariateadjusted for confounders^a^ lifestyle mediators^b^& dental service mediators^c^	
	*N* = 11,059		*N* = 11,059		*N* = 11,059		*N* = 11,059		*N* = 11,059	
	OR (95 % CI)	*p* value	OR (95 % CI)	*p* value	OR (95 % CI)	*p* value	OR (95 % CI)	*p* value	OR (95 % CI)	*p* value
Fillings										
White	1.00		1.00		1.00		1.00		1.00	
Indian	0.27 (0.20-0.37)	<0.001	0.25 (0.17-0.37)	<0.001	0.29 (0.19-0.42)	<0.001	0.30 (0.20-0.46)	<0.001	0.34 (0.22-0.52)	<0.001
Pakistani/Bangladeshi	0.35 (0.24-0.52)	<0.001	0.43 (0.26-0.69)	0.001	0.51 (0.31-0.84)	0.008	0.46 (0.28-0.76)	0.002	0.54 (0.32-0.90)	0.018
Black	0.32 (0.22-0.47)	<0.001	0.33 (0.20-0.52)	<0.001	0.37 (0.23-0.59)	<0.001	0.36 (0.23-0.59)	<0.001	0.42 (0.26-0.69)	0.001
Dental extraction										
White	1.00		1.00		1.00		1.00		1.00	
Indian	0.27 (0.20-0.36)	<0.001	0.33 (0.23-0.47)	<0.001	0.39 (0.28-0.56)	<0.001	0.34 (0.24-0.48)	<0.001	0.41 (0.28-0.58)	<0.001
Pakistani/Bangladeshi	0.29 (0.20-0.41)	<0.001	0.41 (0.26-0.63)	<0.001	0.49 (0.32-0.76)	0.001	0.40 (0.26-0.62)	<0.001	0.48 (0.31-0.75)	0.001
Black	0.44 (0.32-0.62)	<0.001	0.60 (0.42-0.87)	0.007	0.75 (0.52-1.09)	0.135	0.63 (0.43-0.91)	0.015	0.78 (0.53-1.14)	0.197
Less than 20 teeth										
White	1.00		1.00		1.00		1.00		1.00	
Indian	0.24 (0.13-0.42)	<0.001	0.31 (0.16-0.59)	<0.001	0.33 (0.18-0.63)	0.001	0.27 (0.14-0.52)	<0.001	0.30 (0.16-0.58)	<0.001
Pakistani/Bangladeshi	0.15 (0.06-0.35)	<0.001	0.22 (0.08-0.57)	0.002	0.26 (0.10-0.66)	0.005	0.21 (0.08-0.57)	0.002	0.26 (0.10-0.68)	0.001
Black	0.42 (0.25-0.69)	0.001	0.66 (0.38-1.15)	0.142	0.81 (0.47-1.42)	0.469	0.62 (0.35-1.11)	0.108	0.78 (0.44-1.38)	0.197

OR odds ratio; CI confidence interval; N number

^a^age, sex, educational qualifications, housing tenure, area socioeconomic deprivation quintile and area of residence (mid-level output area)

^b^consumption of sweets, cakes and fizzy juices, sugar added to hot drinks, smoking status, frequency of teeth cleaning, use of other dental hygiene products

^c^frequency of dental appointments, type of dental service provider, ever had scale and polish

Odds ratios are from imputed data and are weighted using the ADHS provided weights

After adjusting for the potential confounders, Black participants remained significantly less likely, than White participants, to report fillings and dental extractions (Table 5). The association with less than 20 teeth was no longer statistically significant. Adjustment for use of dental services attenuated the associations with filling and dental extractions but they remained statistically significant. Following adjustment for potential lifestyle mediators, the reduced risk of fillings and extractions was attenuated further and the latter became non significant.

Of the 6,327 eligible dentate participants who underwent the dental examination, 5,909 were White, 319 South Asian (170 Indian, 108 Pakistani or Bangladeshi and 41 other Asian) and 99 Black. In the univariate analysis of the 6,228 White or South Asian participants, the lower risk of clinical evidence of caries among South Asian participants did not reach statistical significance (odds ratio (OR) 0.76, 95 % confidence interval (CI) 0.39-1.47, *p* = 0.411); nor did it following adjustment for potential confounders (adjusted OR 0.62, 95 % CI 0.31-1.25, *p* = 0.182). Addition of lifestyle mediators to the model had little effect but further addition of dental service mediators resulted in a statistically significant lower risk of caries among South Asians (adjusted OR 0.42, 95 % CI 0.20-0.89, *p* = 0.023). In the univariate analysis, there was no significant difference in the risk of having ≥6 periodontal pockets (OR 1.03, 95 % CI 0.63, 1.66) and this remained the case after adjusting for potential confounders as well as for the mediating effects of lifestyle. Further adjustment for dental service mediators increased the association but it remained statistically non-significant (adjusted OR 1.27, 95 % CI 0.75-2.15, *p* = 0.382).

## Discussion

Within the UK, people who belonged to non-White groups were less likely to report fillings, dental extractions, and fewer than 20 teeth. These differences were not explained by dental hygiene and dental services, which were generally used less in non-White groups. Non-White groups were more likely to add sugar to drinks or consume fizzy drinks but they were less likely to consume sweets and cakes. The latter appeared to explain most of the reduced risk in Black participants and some of it in South Asian participants. Dental examination of a sub-group demonstrated fewer dental caries among South Asian participants. Thus, our findings show that non-White groups have generally better oral health, defined by the presence of more teeth, and have had correspondingly fewer dental extractions.

Our findings are consistent with previous studies that have reported little impact of dental services on reducing dental caries [27], and inverse relationships between use of dental services and adverse dental health outcomes [28]. A previous USA study on hypothetical patient preference suggested that Black individuals would be more likely to opt for dental extraction than further root canal restorative treatment; mainly explained by preference, treatment acceptability and ability to afford treatment [29]. In a South American study that presented dentists with hypothetical patient scenarios, the dentists were more likely to choose dental extraction for Black, than White, patients irrespective of their disease severity, lifestyle behaviours or personal choice [30]. Other studies have confirmed that the Black population in the USA and Brazil has more dental caries, more dental extractions and fewer teeth [3, 31]. Our findings suggested that British Black participants had significantly fewer fillings and fewer dental extractions; although the latter did not reach statistical significance. These findings do not necessarily conflict with the American findings since fewer fillings and extractions may reflect poorer access to, or use of, dental services among British Black people, rather than better dental health. We were unable to include Black participants in the sub-group analysis of dental caries, due to lack of statistical power. However, Black participants were significantly more likely, than White, to report poor or very poor oral health suggesting that fewer fillings may reflect unmet need rather than less need. However, further studies are required to ascertain whether this is true.

In 1999, Newton et al. published the first study to demonstrate superior oral health among ethnic minority groups in the UK [26]. These findings have since been corroborated by other UK studies of adults [32–34], but have been refuted by some studies conducted on children and adults in the UK and other countries. Conway et al. demonstrated higher rates of caries among UK Pakistani children, compared with White [22]. Selikowitz and Holst studied the periodontal health of Pakistani people living in Norway and showed a higher prevalence of plaque, sub-gingival calculus and gingival bleeding [35]. Similarly, a study in Singapore demonstrated that Indian residents had worse periodontal health than either Chinese or Malay residents [36].

Overall, smoking prevalence is lower in ethnic minority groups [37]. However, this masks large sex differences. In White populations, smoking prevalence is comparable in men and women [38, 39]. South Asian men have a higher prevalence of smoking than White men [37]. In contrast, the self-reported prevalence of smoking among South Asian women is low [38, 39]. However, these figures do not take account of chewing tobacco which is much more common among South Asian people, including women [40]. In a study by Croucher et al., only 4 % of Bangladeshi women smoked cigarettes but 49 % chewed paan quid with tobacco [41]. Therefore, questionnaires that focus on cigarette consumption are likely to underestimate the use of tobacco products in South Asian study participants.

Williams et al. demonstrated lower levels of dental knowledge among Asian parents compared with White (OR 0.43 95 % CI 0.27-0.70, *p* < 0.05) [42]. Studies conducted in the 1980’s showed that British Asian women were more likely to rub around their mouth with their fingers than use a toothbrush, with some not cleaning their teeth at all [43, 44]. Practices such as the use of chewing sticks, home-made or imported dentifrices were also common. However, it is unclear whether these findings are still relevant.

Risk of caries is increased if the condition of existing fillings, restorations, orthodontic appliances and partial dentures is poorly maintained [45, 46]. Within the ADHS, more than 50 % of participants from ethnic minority groups visited the dentist every six months and 72 % had visited the dentist within the last year. However, a significant proportion of participants in these groups were not undergoing regular dental clinic visits and ethnic minority groups were more likely to restrict dental visits to when symptoms occurred. Our findings are consistent with previous studies on Bangladeshi adults living in the United Kingdom, which showed that 25 % of adults [32] and 58 % of adult women [47] had never visited a dentist. These studies have also highlighted unmet treatment needs in ethnic minority groups with 80 % of Asian adults living in Southampton found to require dental treatment, but only 38.5 % of them perceiving any need [32]. This is consistent with our finding that Pakistani/Banglasdeshi respondents were more likely to report poor oral health but less likely to report having had dental procedures such as fillings and extractions.

In a focus group, conducted by Croucher and Sohanpal [48], members of ethnic minority groups reported difficulties in obtaining dental appointments and longer waiting times. Other barriers identified to using dental services have included language, cultural beliefs and affordability [49]. In a study of Bangladeshi medical care users in the UK [50], language problems were reported by 73 %, with more women facing difficulties than men. Nearly 68 % required an interpreter; resulting in a preference for evening appointments when family members could attend. Indian and Pakistani study participants felt that their inability to explain their dental problems might prolong their dental treatment, thereby increasing treatment costs [50]. In a number of studies, actual cost of dental treatment, or fear of cost, have been reported as major barriers to members of ethnic minority groups accessing dental services [32, 33, 47–50]. Ethnic minority groups perceive dental treatment to be expensive, lacked clarity about the individual treatment fees and reported difficulties in finding a dentist [48]. Other problems reported include fear of dental treatment [32, 48, 49], difficulty obtaining time off work [32], cultural misunderstandings [49], and concerns about hygiene in the dental surgery [49].

The ADHS provided data on a large sample; representative of the general population of England, Wales and Northern Ireland. Whilst the majority of participants were White, there were still sufficient numbers in the main ethnic groups to permit comparisons between ethnic groups within the same study. The survey provided information on a number of potential confounders and mediators, including socio-demographic information, diet, lifestyle, personal dental hygiene and use of dental services, enabling us to explore possible reasons for the observed ethnic differences. We were also able to adjust for both individual level measures of socioeconomic deprivation and an area-based measure. Self-reported oral health was corroborated by clinical examination in the dentate sub-group of participants.

As with all cross-sectional studies, it is impossible to establish temporal relationships. Also, behaviour may change over time and current lifestyle and use of dental services may not equate to those prior to the development of oral health problems. For example, increased use of dental services may be a result of dental problems and, therefore reflect reverse causation. We adjusted for potential confounders but, in common with all observational studies, residual confounding is possible. Whilst overcrowding is a good proxy measure of deprivation among White populations, cultural differences in the acceptability of living with extended families make it a poorer measure of differences in socioeconomic status between ethnic groups. Therefore, the number of household members was not included as a covariate in the models.

As a secondary data study, our analyses were limited to the data and definitions available to us. The ADHS adopted the usual practice in UK surveys and epidemiological studies of using self classification of ethnic group. This is, arguably, a strength since self-identified ethnicity is more likely to reflect health beliefs and behaviours. Both ethnicity and migration influence oral health. Immigrants arrive in the UK with different lifestyle behaviours which, due to assimilation, gradually converge with those of their recipient country over time [50]. However, we were unable to differentiate between the effects of ethnicity and migration since the ADHS collected no information on length of residence in the UK, personal or parental place of birth. Similarly, no information was available on language, religion or beliefs. However, the Survey did provide data on lifestyle risk factors, use of dental services and personal dental hygiene practice all of which are influenced by religion, culture and beliefs and, therefore, ethnicity [51–53]. We also had information on area- and individual-level socioeconomic status which are important confounders in any studies of ethnic differences. However, our study was insufficiently powered for us to investigate area-level differences in outcomes. Therefore, our results may only apply to metropolitan areas. For the three main outcomes, we were able to include all participants and were, therefore, able to include Indian, Pakistani/Bangladeshi and Black participants as three separate groups. Because the clinical examination was undertaken on a sub-group, statistical power was reduced and we had to combine the Indian and Pakistani/Bangladeshi groups into a single, South Asian, group. Since there are some important differences between the three groups in terms of religion, culture, socioeconomic status and education, care should be heeded in interpreting the findings of the sub-group analysis.

## Conclusions

In conclusion, in spite of generally lower use of dental hygiene and preventative dental services, South Asian and Black participants were less likely to report fillings, tooth extractions and tooth loss. This may reflect genuinely better oral health especially since some of these differences could be explained by lower consumption of cakes and sweets. The Survey lacked detailed information on the amount and frequency of sugar consumption; therefore, it is possible that dietary differences may explain all, rather than some, of the ethnic differences. These findings suggest that dietary sugar may not only be the major driver of overall oral health, but may also contribute to ethnic differences in oral health. They also reinforce the need for the population as a whole, and White groups in particular, to reduce dietary sugar consumption if we are to reduce the burden of oral diseases.

However, it should also be noted that the fewer dental interventions reported by Pakistani/Bangladeshi and Black participants was in spite of these groups being more likely, than White, to rate their oral health as poor; suggesting the possibility of some unmet need. Service providers should work with community leaders to ensure that these groups have good access to, and make regular use of, dental services.

## Abbreviations

ADHS, Adult Dental Health Survey; CI, confidence interval; IMD, Index of Multiple Deprivation; N, number; NHS, National Health Service; OR, odds ratio; ROC, receiver operating characteristic; UK, United Kingdom; USA, United States of America
